# Molecular Mechanisms of the Anti-Obesity and Anti-Diabetic Properties of Flavonoids

**DOI:** 10.3390/ijms17040569

**Published:** 2016-04-15

**Authors:** Mohammed Kawser Hossain, Ahmed Abdal Dayem, Jihae Han, Yingfu Yin, Kyeongseok Kim, Subbroto Kumar Saha, Gwang-Mo Yang, Hye Yeon Choi, Ssang-Goo Cho

**Affiliations:** Department of Animal Biotechnology, Animal Resources Research Center, Incurable Disease Animal Model and Stem Cell Institute (IDASI), Konkuk University, Gwangjin-gu, Seoul 05029, Korea; kawsersau07@gmail.com (M.K.H.); ahmed_morsy86@yahoo.com (A.A.D.); hjh6517@naver.com (J.H.); yfy_21@hotmail.com (Y.Y.); proproggs@naver.com (K.K.); subbroto@konkuk.ac.kr (S.K.S.); slayersgod@nate.com (G.-M.Y.); hyeon.choi24@gmail.com (H.Y.C.)

**Keywords:** obesity, diabetes, flavonoids, anti-obesity, anti-diabetic, molecular mechanism

## Abstract

Obesity and diabetes are the most prevailing health concerns worldwide and their incidence is increasing at a high rate, resulting in enormous social costs. Obesity is a complex disease commonly accompanied by insulin resistance and increases in oxidative stress and inflammatory marker expression, leading to augmented fat mass in the body. Diabetes mellitus (DM) is a metabolic disorder characterized by the destruction of pancreatic β cells or diminished insulin secretion and action insulin. Obesity causes the development of metabolic disorders such as DM, hypertension, cardiovascular diseases, and inflammation-based pathologies. Flavonoids are the secondary metabolites of plants and have 15-carbon skeleton structures containing two phenyl rings and a heterocyclic ring. More than 5000 naturally occurring flavonoids have been reported from various plants and have been found to possess many beneficial effects with advantages over chemical treatments. A number of studies have demonstrated the potential health benefits of natural flavonoids in treating obesity and DM, and show increased bioavailability and action on multiple molecular targets. This review summarizes the current progress in our understanding of the anti-obesity and anti-diabetic potential of natural flavonoids and their molecular mechanisms for preventing and/or treating obesity and diabetes.

## 1. Introduction

Obesity and diabetes mellitus (DM; commonly referred to as diabetes) are important health concerns worldwide; their incidence is increasing at an alarmingly high rate, generating enormous social costs [1]. Obesity is frequently observed among those who live long-term sedentary lifestyles, consume large amounts of fast food, or suffer from genetic diseases. Obesity is a complex disease commonly accompanied by insulin resistance, increased oxidative stress, and enhanced inflammatory marker expression. According to the International Obesity Taskforce, more than 300 million people who have a body mass index greater than 30 kg/m^2^ are categorized as obese. The number of obese-born children of developing countries is increasing, as is the number of obese adults in developed countries [2]. One of three children born in the early current century is expected to develop obesity-related diabetes [3,4].

Obesity causes the development of metabolic disorders such as DM, hypertension, cardiovascular diseases, and inflammation-related pathologies [5]. It is expected that in 20 years nearly 600 million adults will become diabetic because of the high obesity prevalence, aging, high population growth, increase in living standards, increased urbanization, and high-calorie fast food consumption [6]. With the growing prevalence of obesity, the number of type 2 diabetes mellitus (T2DM) cases is proportionally increasing [7]. DM is a group of metabolic disorders characterized by high blood sugar levels over a prolonged period resulting from either destruction or impairment of insulin-secreting pancreatic β cells and insulin action in target tissues [8,9]. DM is one of the fastest increasing metabolic diseases worldwide, causing disabling micro- and macrovascular complications [10]. Prevention and/or treatment of DM involve a healthy diet, physical exercise, and maintaining a normal body weight. Consuming healthy foods is particularly important for people with DM. The causes of obesity involve diet, physical inactivity, metabolism, genes, and the socio-cultural environment [11]. Although numerous commercial drugs are available for treating obesity and diabetes, many of these are unavailable to a large number of sufferers and can cause adverse effects. The utilization of medicinal plants and their phytochemicals for treating obesity and diabetes is not only a priority for developing safer alternatives to pharmaceuticals, which transitorily lower blood glucose and prevent high blood pressure and cardiovascular disease, but also enhance the antioxidant system, insulin action, and secretion [12]. Therefore, identifying dietary constituents that can regulate body fat deposition and blood glucose levels is very important.

Flavonoids or bioflavonoids are named from the Latin word *flavus*, meaning yellow, and are ubiquitous in plants; these compounds are the most abundant polyphenolic compounds in human diet [13,14]. They are secondary metabolites of plants and fungi and have a 15-carbon skeleton containing two phenyl rings and a heterocyclic ring. More than 5000 naturally occurring flavonoids have been reported in various plants; these flavonoids show many beneficial effects with advantages over chemical treatments. A number of studies have demonstrated the potential health benefits of natural flavonoids against obesity and DM. This review summarizes the current progress in the study of the anti-obesity and anti-diabetic potential of natural flavonoids and their molecular mechanisms for preventing and/or treating obesity and diabetes.

## 2. Causes of Obesity and Diabetes and the Related Patho-Physiology

**Figure 1:**
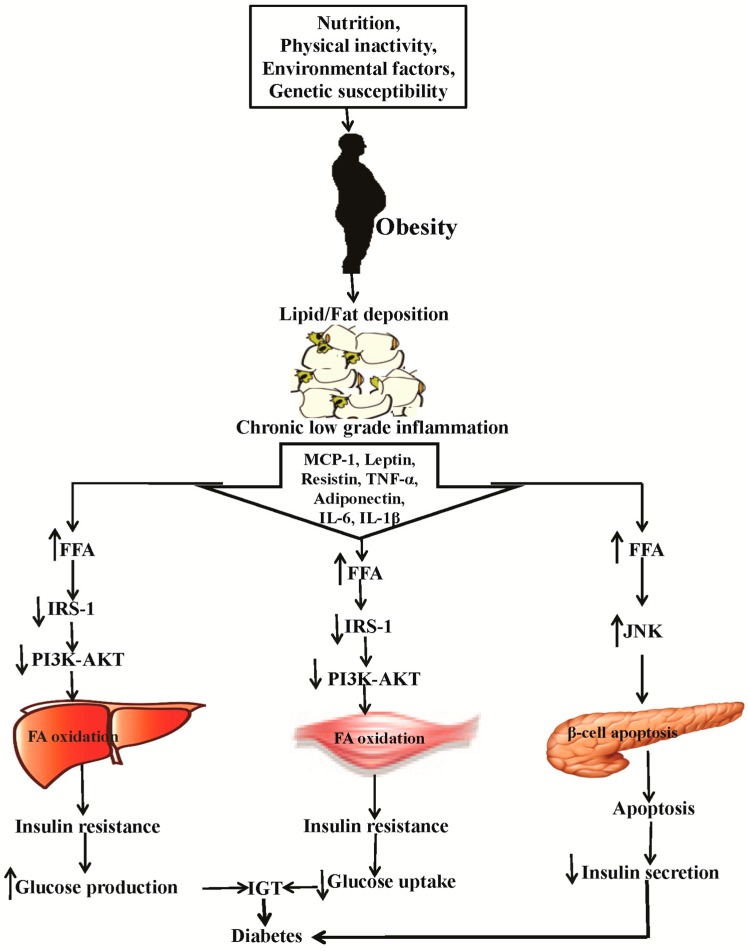
Schematic diagram of the link between obesity and diabetes as well as their effects in skeletal muscle, liver, and pancreas for stimulating different inflammatory cytokines, metabolic enzymes, and signaling pathways. Nutrition, physical inactivity, environmental factors, and genetic susceptibility cause obesity and fat deposition that initiates chronic low-grade inflammation to release MCP-1, leptin, resistin, TNF-α, adiponectin, IL-6, and IL-1β. Chronic inflammation leads to increased secretion of FFA from the liver, skeletal muscles, and pancreas. Increased FFA reduces the expression of IRS-1 and PI3K-AKT in the liver and skeletal muscles and increased JNK expression in the pancreas, ultimately causing insulin resistance in the liver and muscle and increasing apoptosis in the pancreas. Insulin resistance causes glucose production increase and glucose uptake decrease, and insulin secretion decreases because of increased apoptosis of pancreatic β cells. MCP-1: monocyte-chemo-attractant protein-1 [19]; TNF-α: tumor necrosis factor α [21]; IL-6: interleukin-6 [19]; IL-1β: interleukin 1 β [19]; FFA: free fatty acid [23]; IRS1: insulin receptor substrate 1 [24,25]; PI3K: phosphatidylinositol 3-kinase [24,25]; AKT: serine/threonine kinase [24,25]; JNK: c-Jun N-terminal kinase [26] FA: fatty acid [23]; IGT: impaired glucose tolerance [23]. (↓) Decrease, (↑) Increase.

Obesity is associated with the occurrence of low-level chronic inflammation, demonstrating a close link between metabolism and immunity [15,16]. Fat cells known as adipocytes vigorously secrete a mixture of products that link obesity and diabetes (Figure 1). Adipose tissues composed of adipocytes and stromal vascular cells include diverse cell types such as preadipocytes, numerous immune cells, endothelial cells, and fibroblasts. Macrophage infiltration is particularly prominent in the adipose tissue of obese individuals [17,18]. The adipose tissue-derived monocyte-chemo-attractant protein-1 (MCP-1) is a CC chemokine (or β-chemokine), exhibiting chemotactic properties in inflammatory cells, which are key factors for inducing macrophage infiltration into adipose tissue. MCP-1 released by adipocytes is found at high levels in obese mice compared to in non-obese mice, and its levels are distinctly increased when adipocytes are co-cultured with macrophages [19,20,21]. MCP-1 triggers macrophage infiltration into adipose tissue and the subsequent release of inflammatory mediator tumor necrosis factor-alpha (TNF-α) [21], which hampers insulin signaling and stimulates fatty acid lipolysis in adipocytes. TNF-α and other pro-inflammatory cytokines including interleukin-6 (IL-6), interleukin-1 β (IL-1β), and C-reactive protein are involved in low-grade chronic inflammation and insulin resistance [19,22]. Inflammatory cytokines were reported to inhibit triglyceride synthesis by downregulating peroxisome proliferator-associated receptor γ (PPARγ) and its target gene, plasma lipoprotein lipase (LPL), as well as the glucose transporter, glucose transport type 4 (GLUT4) [19,22]. Simultaneously, TNF-α reduces insulin-mediated attenuation of lipolysis, downregulates the lipid droplet-associated protein perilipin (PLIN), and enhances the cAMP pool, all of which increase free fatty acid (FFA) release [23]. Increased FFA reduces the expression of IRS-1, impairs the activation of PI3K-AKT [24,25] signaling in the liver and skeletal muscles, and increases the expression of JNK signaling in the pancreas [26]. Ultimately, the reduced expression of PI3K-AKT causes insulin resistance in the liver and skeletal muscles, and the increased expression of JNK aggravates apoptosis in the pancreas. Insulin resistance causes an increase in glucose production and a decrease in glucose uptake, leading to hyper-insulinemia. Increased apoptosis of pancreatic β cells results in a decrease of insulin secretion. Consequently, insulin resistance and pancreatic β cell apoptosis lead to diabetes [23].

## 3. Flavonoids: Classification and Their Biological Properties

**Figure 2:**
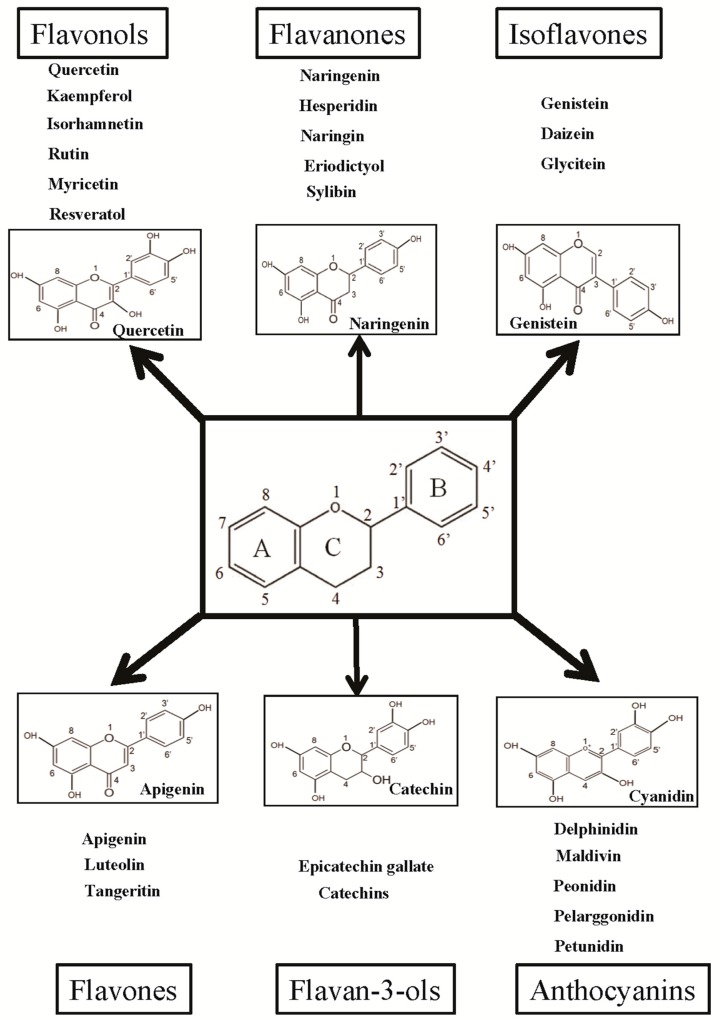
Classification and example of flavonoids and their chemical structures. Flavonoids are classified into six groups, including flavonol, flavanone, isoflavone, flavone, flavan-3-ols, and anthocyanin. Chemical structures of each of the six classes of flavonoids are shown as examples, including isorhamnetin for flavonol, naringin for flavanone, daizein for isoflavone, apigenin for flavone, catechin for flavov-3-ols, and cyanidin for anthocyanins.

**Figure 3:**
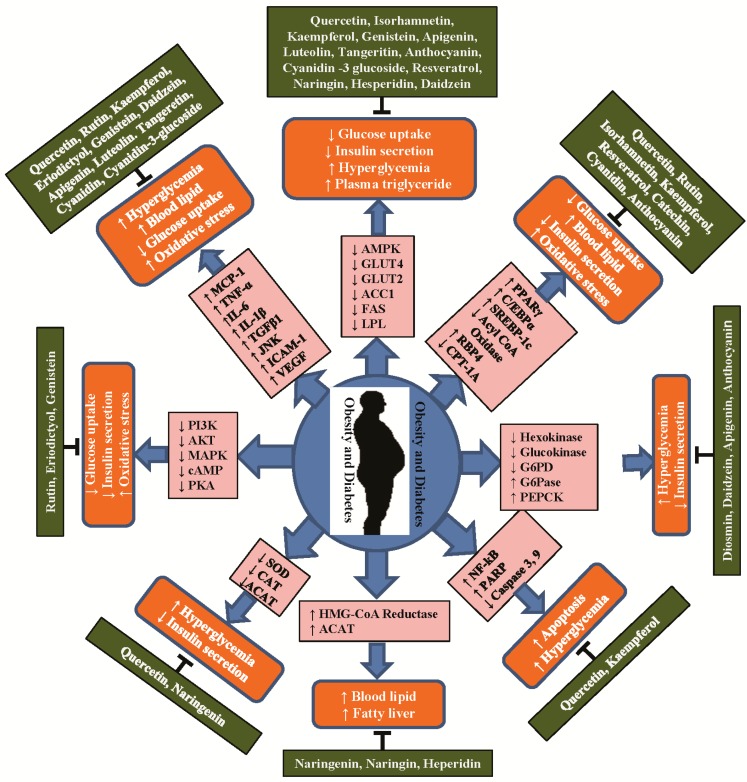
Schematic presentation of molecular functions of different flavonoids with anti-obesity and anti-diabetic effects. Obesity and diabetes stimulate increased or decreased production of inflammatory cytokines, expression of different metabolites, and intracellular cell signaling. Flavonoids showed anti-obesity and anti-diabetic effects by activating or inhibiting different cytokines, enzymes, and metabolites to prevent inflammation, oxidative stress, and metabolism to protect against obesity and diabetes. MCP-1: monocyte-chemo-attractant protein-1; TNF-α: tumor necrosis factor alpha; IL-6: interleukin-6; IL-1β: interleukin 1 beta; FFA: free fatty acid, IRS1: insulin receptor substrate 1; PI3K: phosphatidylinositol 3-kinase; AKT: serine/threonine kinase; FA: fatty acid; IGT: impaired glucose tolerance; PARP: poly(ADP-ribose) polymerase; BCl-2: B-cell lymphoma 2; Bax: Bcl-2-associated X protein; Bak: Bcl-2 homologous antagonist/killer; Caspase 3: cysteine-dependent aspartate-directed proteases 3; PPAR γ: peroxisomal proliferator-activated receptor gamma; SREBP1c: sterol regulatory element binding protein-1c; LPL: lipo protein lipase; AMPK: 5′ adenosine monophosphate-activated protein kinase; HOMA-IR: homeostatic model assessment for insulin resistance; HbA1c: hemoglobin A1c; GLUT4: glucose transporter 4; G6PDH: glucose-6-phosphate dehydrogenase; HMG-CoA: 3-hydroxy-3-methylglutaryl-coenzyme; ACAT: acyl CoA: cholesterol acyltransferase; G6pase: glucose-6-phosphatase; cAMP: cyclic adenosine monophosphate; PKA: protein kinase A. (↓) Decrease, (↑) Increase.

**Figure 4:**
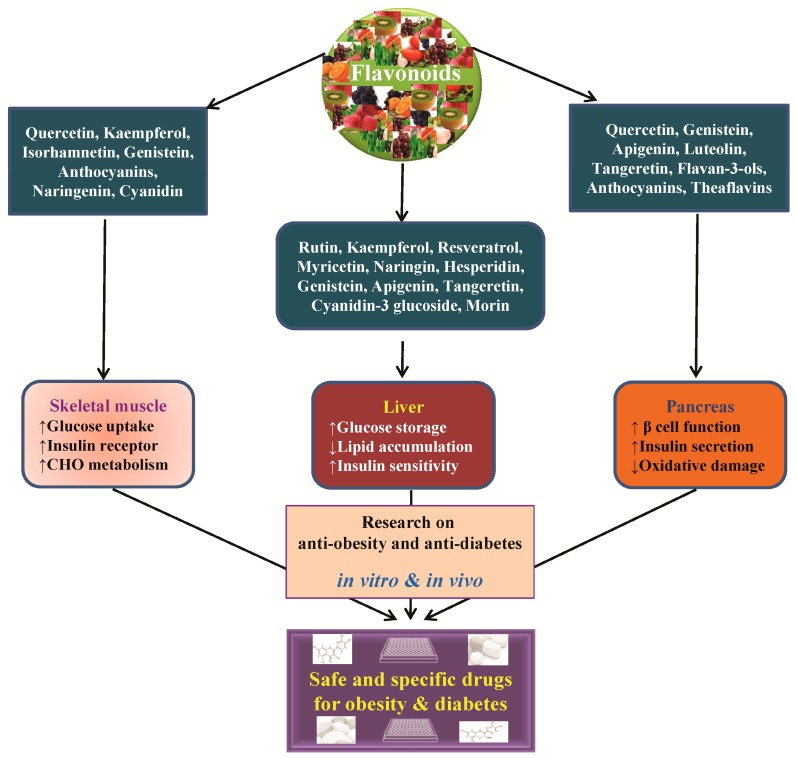
Graphical presentation of anti-obesity and anti-diabetes effect of flavonoids and their subsequent effects in skeletal muscles, liver, and pancreas to induce glucose uptake, increase insulin secretion, and reduce oxidative damage and lipid accumulation. Research on the molecular action of flavonoids would help in developing new strategies for discovery of safe and specific anti-obesity and anti-diabetic drugs. CHO: Carbohydrate. (↑) Increase, (↓) Decrease.

Over 5000 different flavonoids have been isolated and identified from plant sources; these compounds are extensively distributed in the plant kingdom, particularly in photosynthesizing plant cells [27,28]. Flavonoids are a diverse group of polyphenolic compounds primarily known as the pigments responsible for producing the many colors present in flowers, fruit, and leaves. These polyphenolic compounds were well known for their medicinal properties in health long before they were more closely evaluated in studies. Over the last 20 years, a significant amount of research has focused on polyphenol compounds of plant origin because of their potential benefits in human health [29]. Flavonoids are composed of a 15-carbon (C6–C3–C6) skeleton and two benzene rings joined by a linear 3-carbon chain. Flavonoids can be divided into multiple subgroups according to the substitution patterns of the ring C, and flavonoids within the same class can be differentiated by the substitution of A and B [27,30,31]. There are six major subgroups of flavonoids, including flavonols (including quercetin, kaempferol, and myricetin), flavanones (including eriodictyol, hesperetin, and naringenin), isoflavonoids (including daidzein, genistein, and glycitein), flavones (including apigenin and luteolin), flavans-3-ol (including catechin), and anthocyanins (including cyanidin, delphinidin, malvidin, pelargonidin, peonidin, and petunidin) (Figure 2). A number of clinical and research studies have suggested that flavonoids have positive effects in the treatment, prevention, and alleviation of various viral diseases [32,33], degenerative diseases, such as cardiovascular diseases, cancers [34], diabetes [35], obesity, and other age-related diseases [27,30,36,37,38]. Flavonoids can function as antioxidants to prevent diseases by modulating oxidative stresses in the body. In our laboratory, we carried out several *in vitro* and *in vivo* studies to examine these antioxidant [34,39], anti-cancer [40,41,42], and antiviral activities [32,33] of specific flavonoids. It has been reported that free radicals such as reactive oxygen and nitrogen species, which are byproducts of cell metabolism in humans, can cause various life-threatening diseases such as coronary heart diseases, obesity, T2DM, and cancers [43,44]. Thus, flavonoids act as antioxidants against various diseases by neutralizing the effects of reactive oxygen and nitrogen species. Numerous *in vitro* cell and *in vivo* animal studies support the beneficial effects of dietary flavonoids on glucose homeostasis for the prevention and treatment of obesity and diabetes (Figure 3 and Table 1). Flavonoids regulate carbohydrate digestion, adipose deposition, insulin release, and glucose uptake in insulin-responsive tissues through numerous cell-signaling pathways (Figure 4).

## 4. Anti-Obesity and Anti-Diabetic Properties of Flavonoids and Their Molecular Functions

### 4.1. Flavonol

Flavonols are the most abundant flavonoids in the plant kingdom. The main dietetic flavonols include quercetin, kaempferol, isorhamnetin, fisetin, and myricetin [85]. Quercetin is one of the most plentiful flavonoids in human dietary nutrition and forms the skeletons of other flavonoids, such as hesperidin, naringenin, and rutin. Quercetin is found in various foods such as apples, berries, red onions, grapes, cherries, broccoli, pepper, coriander, citrus fruits, and tea (*Camellia sinensis*), and at high concentrations in capers and the large, edible, white flowered plants of the parsley family known as lavages (*Levisticum officinale*). Quercetin has a wide range of biological properties such as lowering of blood pressure [45,47], reduction of body weight [47], and amelioration of hyperglycemia-related diseases in animal models and in humans [86,87].

Quercetin supplementation was reported to reduce blood pressure in hypertensive patients [45]. Its antioxidant activity may also suppress the elevation of blood pressure in diet-induced obesity rat models [47]. Quercetin was reported to stimulate apoptosis in 3T3-L1 preadipocytes by decreasing the mitochondria membrane potential, downregulating expression of B-cell lymphoma 2 (Bcl-2) and poly(ADP-ribose) polymerase (PARP), and activating Bcl-2 homologous antagonist/killer (Bak), Bcl-2-associated X protein (Bax), and cysteine-dependent aspartate-directed proteases 3 (caspase 3) [88]. In growing preadipocytes, quercetin extensively decreased the expression of LPL, sterol regulatory element-binding protein 1c (SREBP1c), and PPARγ, a key adipogenic transcription factor [46,89]. Quercetin caused dose- and time-dependent increases in lipolysis in rat adipocytes, synergistically with epinephrine (also known as adrenalin or adrenaline), which plays a pivotal role in the fight-or-flight response by augmenting blood flow to the muscles, increasing cardiac output, dilating the pupils, and increasing blood sugar [90]. Triglyceride breakdown and fatty acid and glycerol release are vital for the control of energy homeostasis in adipocytes.

Berry extract rich in quercetin was shown to induce the insulin-independent 5′ adenosine monophosphate-activated protein kinase (AMPK) signaling pathway in muscle cells and slow adenosine diphosphate-stimulated oxygen consumption in isolated mitochondria [91]. Notably, this mechanism is analogous to that of metformin (*N*,*N*-dimethylimidodicarbonimidic diamide), the first-line medication used to treat T2DM. Additionally, quercetin derivatives such as isoquercetin (quercetin-3-*O*-glucoside) and hyperoside (quercetin-3-*O*-galactoside) as well as quercetin aglycone, which can be isolated from berry extract, may also improve insulin-independent glucose uptake and stimulate AMPK in muscle cells. Therefore, quercetin and its derivatives are thought to be the major bioactive components in berry that activate AMPK and stimulate glucose uptake in muscle cells. The anti-diabetic effect of quercetin was also investigated in streptozotocin (STZ)-induced diabetic mice; treatment of quercetin resulted in the reduction of hyperglycemia-stimulating GLUT4 and glucokinase, increased liver glucose uptake, and decreased hepatic glycogenolysis and gluconeogenesis [48,92,93]. Dietary supplementation of 0.5% quercetin in the diet for two weeks enhanced serum insulin concentrations and lowered blood glucose in STZ-induced diabetic mice. Moreover, a diet supplemented with quercetin caused upregulation of the expression of genes associated with cell proliferation and survival in the liver [92]. Intraperitoneal (IP) injection of quercetin into STZ-induced diabetic rats led to decreased hyperglycemia and improved glucose tolerance, increasing hepatic glucokinase activity and reducing plasma cholesterol and triglycerides [94]. Additionally, supplementation of 0.04% quercetin in the diet decreased blood glucose and improved insulin resistance in obese diabetic mice [95]. Another study showed that supplementation with quercetin at 30 mg/kg body weight (approximately equivalent to 0.045% quercetin in the diet) per day for six weeks in 6-week-old male Wistar rats fed a high-fat high-sucrose diet, significantly reduced basal levels of glucose and insulin [96]. A number of studies demonstrated the direct action of quercetin on insulin-secreting β cells [97]. Both quercetin and its glycoside derivatives improved glucose-stimulated insulin secretion and repressed oxidative stress and nitric oxide accumulation by regulating NF-κB and ERK 1/2 to protect INS1 cells and clonal pancreatic β cells. Taken together, quercetin is an effective biomolecule that acts on obesity and diabetes by inhibiting the digestion of intestinal starch and hepatic glucose production, increasing glucose uptake in the skeletal muscle, and protecting against pancreatic islet damage.

Rutin (a glycosylated quercetin, also known as rutoside, quercetin-3-*O*-rutinoside, and sophorin), which can be normally extracted from natural plant sources such as buckwheat, oranges, grapes, lemons, limes, peaches, and berries, was also reported to have anti-obesity and anti-diabetic functions [98,99]. Diabetic mice fed with 100 mg/kg rutin in the diet showed significant reductions in plasma glucose levels and increased insulin levels along with the reestablishment of glycogen content and the activities of carbohydrate metabolic enzymes [100]. Rutin was also found to activate liver enzymes linked with the gluconeogenic and lipid metabolic processes. The flavonoid also reduced the levels of fasting blood glucose, blood urea nitrogen, and creatinine and the intensity of oxidative stress, with a significant increase in phosphorylation of mothers against decapentaplegic homolog 7 (SMAD7), an inhibitory SMAD, I-SMAD. SMAD7 belongs to the transforming growth factor β (TGFβ) superfamily of ligands and is a TGFβ type 1 receptor antagonist that blocks the association of the TGFβ type 1 receptor and SMAD2, a receptor-regulated SMAD, R-SMAD. Rutin was shown to influence glucose uptake in the rat soleus muscle through the phosphatidylinositol-4,5-bisphosphate 3-kinase (PI3K) and mitogen-activated protein kinase (MAPK) pathways [101]. Rutin was also reported to reduce the levels of plasma glucose, hemoglobin A1C (HbA1c, a glycated (beta-*N*-1-deoxy fructosyl) hemoglobin), and cytokines, including IL-6 and TNF-α. The flavonoid also led to the reestablishment of antioxidant status and serum lipid profile in STZ-treated diabetic rats fed a high-fat diet (HFD/STZ) [102]. Particularly, rutin can defend against and improve myocardial dysfunction, oxidative stress, apoptosis, and inflammation in the hearts of diabetic rats [103]. A recent report showed that rutin supplementation restored the reduced levels of brain-derived neurotrophic factor (BDNF), nerve growth factor (NGF), and glutathione (GSH) and decreased the level of thiobarbituric acid reactive substances (TBARS), which are formed as a byproduct of lipid peroxidation. Additionally, treatment with rutin in the diabetic retina showed anti-apoptotic activity by decreasing the intensity of caspase 3 and increasing the level of Bcl-2 [104].

Isorhamnetin, an *O*-methylated flavonol, is commonly found in medicinal plants such as *Ginkgo biloba* (known as ginkgo), *Hippophae rhamnoides* (commonly known as sea-buckthorn), and *Oenanthe javanica* (blume, Japanese parsley, Chinese celery, seri in Japanese, or minari in Korean) [105]. It has several biological properties, including anti-diabetic and anti-obesity activities. Oral administration of isorhamnetin at a dose rate of 10 or 20 mg/kg body weight for ten days effectively reduced hyperglycemia and oxidative stress in a STZ-induced model of diabetes. In another study, oral administration of isorhamnetin not only significantly inhibited serum glucose concentration, but also reduced the accumulation of sorbitol in the red blood cells, lenses, and sciatic nerves in STZ-induced diabetic rats [106]. A recent study suggested that isorhamnetin glycosides have anti-diabetic actions and modulate the expression of endoplasmic reticulum stress markers and lipid metabolism [107].

Kaempferol is a member of the flavonol group of flavonoids and is abundant in apple, grape, tomato, tea, potato, broccoli, spinach, and some edible berries [108,109]. Kaempferol extracted from *Bauhinia forficata* leaves reduced hyperglycemia and enhanced glucose uptake in the rat soleus muscle similarly to the action of insulin [110]. *In vitro* results confirmed that kaempferol treatment (10 μM) promoted cell viability, repressed cellular apoptosis, and reduced caspase 3 activities in β cells and human islets continually exposed to hyperglycemic conditions. These defensive effects were related to the improved expression of anti-apoptotic AKT (also known as protein kinase B (PKB)) and Bcl-2 proteins, enhanced cAMP signaling, and increased secretion and synthesis of insulin in β cells [111]. Moreover, kaempferol stimulated glucose uptake in the rat soleus muscle via the PI3K and protein kinase C (PKC) pathways and the synthesis of new glucose transporters [53]. Kaempferol also reduced the expression of TNF-α and IL-1β as well as lipid peroxidation, resulting in improvement of antioxidant defense and body weight gain in diabetic rats [112,113]. Orally administrated kaempferol notably decreased fasting blood glucose and serum HbA1c levels and improved insulin resistance [114]. In liver cells, gene expression analysis showed that kaempferol decreased PPAR-γ and SREBP-1c expression. The anti-obese and anti-diabetic properties of kaempferol were regulated by SREBP-1c and PPAR-γ modulation through AMPK activation [55,114]. The molecular mechanism of the anti-obese and anti-diabetic effects of kaempferol appears to be similar to that of resveratrol, another natural bioactive phytochemical abundant in ground nuts, peanuts, red grapes, and red wine [115,116,117]. Similarly to kaempferol, resveratrol is a potent antioxidant and anti-inflammatory agent [118] and shows a broad range of bioactivities, including the prevention of cancer, diabetes, obesity, and cardiovascular disease [115,116,117]. For the prevention and control of obesity and diabetes, resveratrol was found to regulate the phosphorylation of AMPK to upregulate the fatty acid oxidation and increase glucose uptake via GLUT4 translocation [56]. Moreover, resveratrol suppressed the expression of CCAAT/enhancer-binding protein alpha (C/EBPα) and PPARγ [119,120] and increased fatty acid-binding protein 4 (FABP4) expression [120] in pre-adipocytes, leading to mitochondrial biogenesis and oxidative phosphorylation through the upregulation of the NAD-dependent deacetylase Sirtuin-1 (SIRT1; silent mating type information regulation 2 homolog), thus suppressing lipid accumulation [57,121,122]. Supplementation of resveratrol to the livers of mice fed a high-fat atherogenic diet increased SIRT1 and repressed PPARγ expression and fat accumulation in the livers [121]. Another study showed that treatment with resveratrol slowed PPARγ expression partially by degrading the ubiquitin-dependent proteasome [57]. Resveratrol inhibited fatty acid and triglyceride synthesis, contributing to the lipid-lowering effect [123]. Cell culture studies also showed that resveratrol increased lipolytic activity in adipocytes by inducing cAMP and reducing adipogenesis in isolated human adipocytes [58]. Several animal studies revealed that resveratrol reduces fat depot size and total body fat in HFD and heritably obese rodents [124]. Treatment of rats with 30 mg resveratrol per kg body weight for six weeks fed a hyper-caloric and high-fat diet reduced total adipose tissue [125] and visceral fat and liver mass indices [121]. Additionally, resveratrol reduced blood insulin levels and hyperglycemia in animal models of diabetes [126]. Thus, *in vitro* and *in vivo* studies suggest that kaempferol and resveratrol effectively prevent obesity and diabetes through a diversified mechanism of action.

Myricetin, another flavonol found in teas, wines, berries, fruits, and vegetables, also shows anti-obesity and anti-diabetic properties [127,128]. Myricetin injected intravenously into genetically obese diabetic rats reduced the glucose-insulin index. Treatment with myricetin led to augmentation of GLUT4 expression [129,130] and increased the phosphorylation of AKT and insulin receptor substrate 1 (IRS1) [129,130,131]. Myricetin also stimulated the activity of hepatic glycogen synthase I and glucose-6-phosphate and increased the uptake of glucose in rat adipocytes and boosted insulin-influenced lipogenesis in adipocytes [132]. Supplementation of 0.12% myricetin in mice fed a high-fat high-sugar diet resulted in decreased body weight and improved hypercholesterolemia and hypertriglyceridemia [133], confirming that myricetin can improve insulin secretion and reduce diabetes and obesity.

### 4.2. Flavanones

Naringenin and hesperidin, the two major flavanones that are abundant in citrus fruits such as grape, tomatoes, and oranges, have been reported to possess antioxidant, anti-diabetic, lipid-lowering, anti-atherogenic, and anti-inflammatory activities [61,134,135,136]. Both naringin and naringenin (the aglycone form of naringin) have been extensively studied and have been found to possess anti-obesity and anti-diabetic properties [137,138]. The anti-obesity effect of naringenin was dependent on the reduction in adipose tissue mass and inhibition of preadipocyte proliferation [60]. Naringenin suppressed the proliferation of preadipocytes without showing detrimental effects on subsequent adipogenesis. Moreover, naringenin increased fatty acid oxidation in hepatocytes by enhancing peroxisomal β-oxidation in mice [139]. This compound also remarkably increased the activity of various enzymes required for fatty acid oxidation in hepatocytes, such as acetyl-coenzyme A acetyltransferases (ACAT, also known as thiolase), acyl-coenzyme A oxidase, carnitine *O*-octanoyl transferase (COT, also known as medium-chain/long-chain carnitine acyltransferase), and 3-ketoacyl-coenzyme A [139]. Naringenin included as a 0.1% dietary supplement in rats fed a high-cholesterol diet reduced the cholesterol levels of plasma and triacylglycerol and the cholesterol levels in hepatocytes by decreasing the activity of 3-hydroxy-3-methylglutaryl-coenzyme (HMG-CoA) reductase and ACAT [59]. In 3T3-L1 adipocytes, naringenin repressed glucose uptake [60] and suppressed PI3K and Akt phosphorylation normally induced by insulin, thus regulating insulin-induced GLUT4 translocation [140]. Moreover, naringenin prevented dyslipidemia and improved glucose metabolism by modulating the decrease in blood glucose and lipids independently of fibroblast growth factor 21 (FGR 21) [141]. Taken together, the findings indicate that routine consumption of naringenin impairs glucose uptake in the adipose tissue by exacerbating insulin resistance in susceptible individuals. These antagonistic actions of naringenin on the homeostasis of glucose may depend on an individual’s capacity to absorb and metabolize this flavonoid [60]. Naringin, a flavanone-7-*O*-glycoside between the flavanone naringenin and the disaccharide neohesperidose, also shows biological and pharmacological properties, such as antioxidant, anti-inflammatory, anti-carcinogenic, lipid-lowering, and anti-diabetic effects [62,64,142]. Several studies have demonstrated that in *db*/*db* mice or rats, naringin regulated the plasma lipids in hypercholesterolemic animals fed a HFD [64,138,143]. Diet supplementation with 0.02% naringenin in rats fed a high-fat and high-cholesterol diet for three weeks had no hypolipidemic effect [45,47]. In the livers of *db*/*db* mice, naringin modified the activities of hepatic lipid-metabolizing enzymes and improved plasma lipid metabolism [64]. Furthermore, in T2DM-affected mice, naringin may upregulate hepatic and adipocyte PPARγ and GLUT4 to regulate the expression of hepatic enzymes involved in glycolysis and gluconeogenesis, thereby improving hyperglycemia [64,144]. Daily consumption of naringin consumption decreased plasma low-density lipoprotein (LDL)-cholesterol in hypercholesterolemic individuals [138], suppressed the biosynthesis of hepatic cholesterol, and decreased the levels of plasma lipids and glucose [138,143], supporting that naringin plays a vital role in obesity prevention.

Regarding the lipid-lowering tendency, another flavanone, hesperetin (the aglycone form of hesperidin), lowered the plasma levels of cholesterol and triacylglyceride and the action of the cholesterol biosynthesis rate-limiting enzyme, HMG-CoA reductase, when fed at a 0.02% dietary level to high cholesterol and high-fat-fed rats [145]. Hesperetin also lowered the activity of another key cholesterol-regulating enzyme, ACAT, which is involved in the esterification and absorption of cholesterol. Moreover, hesperetin obstructed cholesterol biosynthesis, resulting in a lower intracellular supply of cholesterol and over-expression of hepatic LDL receptors, as well as increased the clearance of circulating LDL particles [146].

Hesperidin is a flavone glycoside (bound to the disaccharide rutinose) abundant in citrus fruits such as lemons and limes that shows lipid-lowering effects [147]. Hesperidin supplementation to the regular diet regulated the activities of glycolytic and gluconeogenesis enzymes of hepatic glucose metabolism and improved hyperglycemia in *db*/*db*, C57BL6 mice [134,148]. The flavonoid was reported to be beneficial for lowering blood glucose levels by upregulating hepatic glucokinase, PPARγ, and adipocyte GLUT4 [64,149]. This compound is also very effective for advancing the lipid metabolism in *db*/*db* mice by increasing fecal triglyceride excretion and impeding lipid-metabolizing enzymes including glucose-6-phosphate dehydrogenase (G6PDH) and fatty acid synthase [64]. In STZ-induced diabetic rats, hesperidin supplementation also decreased glucose-6-phosphatase (G6Pase), which is a glucose-6-phosphate (G6P)-hydrolyzing enzyme, and increased glucokinase (GK), which is a G6P-generating enzyme, collectively diminishing glucose export via glucose transporter membrane proteins [63]. Hesperidin also reduced plasma and hepatocyte cholesterol levels partially by suppressing hepatic HMG-CoA reductase and ACAT activities, resulting in decreased hypercholesterolemia and atherosclerosis [150,151]. It also increased fecal cholesterol excretion [64]. These coordinated responses of hesperidin supplementation play a significant function in controlling glucose and lipid metabolism in *db*/*db* mice [64]. Taken together, hesperetin and hesperidin have diverse effects on glucose and lipid metabolism and exhibit lipid-lowering activity both *in vitro* and *in vivo*.

Eriodictyol, another flavanone abundant in lemons, also significantly controlled obesity and diabetes [65]. This flavonoid inhibited the adipocyte-specific fatty acid binding protein in 3T3-L1 adipocytes by suppressing PPARγ and increasing the glucose uptake, improving insulin resistance [65]. Eriodictyol also impeded diabetes-related lipid peroxidation by decreasing the levels of TNFα, intercellular adhesion molecule 1 (ICAM-1), and vascular endothelial growth factor (VEGF) [66].

### 4.3. Isoflavones

Isoflavones are another class of flavonoids commonly found in leguminous plants, including soybean and soy products; the major dietary isoflavones are daidzein and genistein, which are present primarily in soy foods [85]. Numerous studies have suggested that isoflavones favorably affect adiposity, glucose homeostasis, insulin secretion, and lipid metabolism [152]. Isoflavones have beneficial effects on major risk factors of cardiovascular disease such as excess body weight, hyperinsulinemia, and hyperlipidemia, which are commonly associated with obesity. A mixture of synthetic daidzein and genistein fed at 23.6 mg/kg body weight per day to Sprague-Dawley rats reduced plasma triglycerides more significantly than in casein-fed rats [67]. Additionally, hamsters fed pure synthetic daidzein (16 mg/kg body weight/day) considerably lowered blood glucose and plasma total cholesterol levels compared to casein-fed rats [153,154]. Moreover, supplementation of 500–1500 ppm genistein with a serum equivalent of approximately 2 μM in the diet showed hypolipidemic effects by decreasing fat-pad weights by 50% in C57/BL6 mice [155]. In C57/BL6 ovariectomized mice, subcutaneous injections of genistein (8–200 mg/kg/day) for 21 days decreased adipose tissue gain [155]. Isoflavones reduced adipose tissue deposition, and *in vitro* studies showed that genistein and daidzein enhanced lipolysis by suppressing 3′,5′-cyclic-AMP phosphodiesterase (cAMP-specific PDE) [156,157]. Furthermore, genistein substantially activated AMPK and acetyl-CoA carboxylase (ACC) in cultures of 3T3-L1 adipocytes and suppressed adipocyte differentiation [71]. Genistein induced intracellular reactive oxygen species (ROS) release, which quickly triggered AMPK and led to apoptosis. Adipocytes treated with genistein readily decreased the protein expressions of PPARs and C/EBP. Genistein supplementation also repressed the incorporation of glucose into lipids and increased the output of fatty acids into the medium in an isolated perfused liver preparation [158]. Hence, in the liver and the adipose tissues, genistein may affect lipid metabolism by disrupting both lipolysis and lipogenesis. In type 1 diabetes mellitus (T1DM) animals, dietary supplementation of genistein led to modulation of glucose metabolism and insulin levels [159,160]. A previous study revealed that genistein had anti-diabetic effects by improving plasma lipids [161], thereby increasing insulin sensitivity [69]. A recent study demonstrated that mice given a soy-supplemented diet (containing approximately 198 ppm daidzein and 286 ppm genistein) from conception through adulthood exhibited an improved lipid profile and glucose metabolism [162]. Soy intake also led to increased phosphorylation of AMPK and favorable metabolic changes, including enhanced mitochondrial biogenesis and glucose uptake in the skeletal muscle [162], with decreased blood glucose, TGFβ 1, and HbA1C levels [70,163]. Indeed, recent findings indicated that isoflavone administration lowered plasma glucose, although insulin sensitivity or the plasma lipid profile was unaffected in obese and diabetic animals [164]. Taken together, the metabolism could be differentially modulated by a mixture of isoflavones, soy protein, or genistein. Supplementation of daidzein or genistein in diet at a dose of 0.02% can suppress the onset of diabetes and enhance glucose homeostasis through stabilization of pancreatic β-cell function in non-obese diabetic (NOD) mice, [165]. Isoflavone supplementation was also associated with suppression of the activities of gluconeogenic enzymes such as phosphoenolpyruvate carboxykinase (PEPCK) and G6Pase, as well as β-oxidation of fatty acids and increased lipogenesis in the liver [165]. It was recently reported that genistein reduced fasting glucose in non-genetic diabetes mice [69]. Consistent with this observation, genistein improved glucose tolerance and hyperglycemia and significantly enhanced islet β-cell proliferation and survival in STZ-induced diabetic mice [166]. Genistein was found to exert its effect on β-cells by modulating multiple signaling pathways, including activation of calmodulin kinase II and Ca^2+^ signaling [167] and suppression of the NF-κB, ERK-1/2, and JAK/STAT pathways [168]. In pancreatic β-cells, genistein-induced stimulation of cAMP/PKA signaling was important for its insulinotropic and mitogenic properties [68,169]. In post-menopausal women with T2DM, daily isoflavone intake (100 mg of aglycones) for one year resulted in improved insulin sensitivity and blood lipid parameters [170]. However, in another study of postmenopausal women with T2DM, consumption of isoflavones (132 mg) for 3 months did not improve plasma A1C, blood glucose, and insulin levels [171]. Although the disparities were likely caused by differences in treatment dosage and duration, many *in vitro* or *in vivo* studies have revealed the anti-obesity and anti-diabetic effects of dietary isoflavones.

### 4.4. Flavones

Flavones are another class of flavonoids found mainly in celery, parsley, and many different herbs. The major dietary flavones include apigenin and luteolin [85]. Plants containing apigenin, such as passionflower and chamomile, have been used as traditional medicines for hundreds of years to treat a variety of diseases. Oral administration of apigenin (0.78 mg/kg body weight) for 10 days was reported to reverse the reduction in hepatic antioxidants in alloxan-induced insulin-dependent diabetic mice, confirming the free-radical scavenging activity [172]. In STZ-induced diabetic rats, intraperitoneal administration of apigenin had a significant anti-hyperglycemic effect [72]. In clonal β-cells, apigenin treatment attenuated 2-deoxy-d-ribose-induced apoptosis through its antioxidant effect by controlling the mitochondrial membrane potential [73]. In human THP-1 monotypic cells, apigenin suppressed TNF-α- and IL-1β-induced activation of NF-κB [173] and, in HepG2 hepatocytes, the flavonoid improved AMPK phosphorylation [56]. Apigenin was 200-fold more potent than metformin, a well-known activator of AMPK. In HepG2 cells exposed to high glucose, apigenin was found to decrease ACC phosphorylation and impede lipid accumulation [56], supporting that apigenin has beneficial effects on dyslipidemia and diabetes by regulating AMPK-dependent energy metabolism.

Another anti-obesity and anti-diabetic flavone, luteolin, is abundant in vegetables and fruits such as onion leaves, cabbage, broccoli, celery, parsley, carrots, peppers, apple skins, and chrysanthemum flowers [174,175,176]. In primary mouse adipose cells and 3T3-L1 adipocytes, luteolin was reported to potentiate insulin action and enhance the expression and transcriptional activation of PPARγ target genes [74]. Luteolin also mediated the beneficial effects on metabolic pathways in insulin resistance and DM pathophysiology by repressing the circulating levels of inflammatory molecules such as MCP-1 and resistin [177]. Additionally, luteolin enhanced insulin release in uric acid-damaged pancreatic β-cells by suppressing the reduction of MAFA, principally via the NF-κB and inducible nitric oxide synthase–nitric oxide (iNOS–NO) signaling pathways [75].

Tangeretin, which is prevalent in citrus fruits, including mandarins and oranges, also showed anti-obesity and anti-diabetic effects. In HFD-induced obese mice, administration of tangeretin (200 mg/kg) led to decreased total cholesterol and blood glucose and regulation of adipocytokines, such as adiponectin, IL-6, leptin, MCP-1, and resistin [76]. In diabetic rats, tangeretin treatment (100 mg/kg) for 30 days significantly reduced plasma glucose levels. In diabetic rats, tangeretin treatment enhanced glycolytic enzymes, leading to control of glucose metabolism in the hepatic tissues [77]. In 3T3-L1adipocytes, tangeretin was found to improve the secretion of insulin-sensitizing factor adiponectin while suppressing the secretion of the insulin receptor substrate factor MCP-1 [178].

### 4.5. Flavan-3-ols

Flavan-3-ols are also referred to as flavanols and are present in various teas, fruits, cocoa, and chocolates [179]. In fruits and cocoa, the most common falavan-3-ols are catechin and epicatechin, while in grapes, teas, and seeds of certain leguminous plants, the main falavan-3-ols are epicatechingallate (ECG), gallocatechin, epigallocatechin (EGC), and epigallocatechin gallate (EGCG). Tea and tea components have anti-obesity or anti-diabetic effects [180,181,182,183]. Regular administration of green tea or EGCG has been shown to be effective for preventing cardiovascular and metabolic diseases [184]. Catechin-enriched green tea enhanced energy expenditure and suppressed dietary lipid absorption [185]. In animal models, catechin and EGCG were studied for their effect to minimize diet-induced obesity by increasing fat oxidation and decreasing leptin levels and energy absorption [179,186]. Overweight or obese men aged 40–65 years who consumed 400 mg capsules of EGCG with the diet twice daily for eight weeks revealed the potential anti-obesity effects of EGCG [187]. In cases of T2DM, the beneficial effects of (−)-catechin in the treatment of obesity-related diseases were also observed, with enhanced insulin-dependent glucose uptake in differentiated adipocytes [79]. The molecular mechanism responsible for stimulating the effect of (−)-catechin on adiponectin expression involved the repression of Kruppel-like factor 7 (KLF7) expression, which regulates the expression of adiponectin and other adipogenesis-related genes, such as PPARγ, leptin, CEBPβ, and aP2 in adipocytes [79]. In mice fed a HFD, treatment with EGCG attenuated hyperlipidemia and fatty liver [80] and in human HepG2 cells, ECG and EGCG diminished the accumulation of hepatic lipids and suppressed fatty acid synthase and acetyl Co-A carboxylase 1 (ACC1) *in vitro* and *in vivo* [186,188]. ACC1 is located in the cytosol and slows the β-oxidation of fatty acids through malonyl-CoA formation to inhibit fatty acid transport, which is mediated by mitochondrial carnitine palmitoyl transferase (CPT1, also known as carnitine acyltransferase I) [189]. ACC1, caspase 3, cyclin-dependent kinase 2 (Cdk2), and AMPK were reported to be involved in flavan-3-ol-mediated modulation of obesity- and diabetes-related apoptosis and ROS generation [190]. Numerous studies reported the anti-diabetic effects of flavan-3-ols in animal and cell culture studies. In rat insulinoma-m5F cells under glucose-induced toxicity, treatment of flavan-3-ols led to improvement in the insulin secretory function and viability of β-cells through increased expression of insulin receptor substrate 2 (IRS2), AKT, forkhead box protein O1 (FOXO1), and pancreatic duodenal homeobox-1 (PDX-1) [191]. Additionally, flavan-3-ols enhanced mitochondrial action by increasing the quantity and entire efficiency of mitochondria [191]. Insulin function and the insulin-mediated signaling pathway was significantly modulated by IRS2 and deletion of the IRS protein led to T2DM [192]. The important factors in IRS signaling pathway were AKT, PDX-1, and FOXO1 [193]. PDX-1 could regulate the pancreas development and function and FOXO1 was reported to induce NeuroD and MAFA expression, an important function in modulation of β-cell proliferation and apoptosis [194].

### 4.6. Anthocyanidins and Other Flavonoids

Anthocyanidins are another class of flavonoids widely distributed in the human diet in fruits, vegetables, berries, and red wine [82]. Considerable attention has been given to anthocyanins because of their potential health benefits including anti-inflammatory, antioxidant, anti-obesity, and anti-diabetic effects [195]. More than 635 anthocyanin compounds have been identified; the most prevalent of these compounds include cyanidin, delphinidin, malvidin, peonidin, pelargonidin, and petunidin [196]. The therapeutic implications of cyanidin 3-glucoside include anti-obesity and anti-diabetes capacities [197]. In an HFD-induced rat model, supplementation of the flavonoid repressed body weight increases, decreased white and brown adipose tissue weights, and enhanced hyperinsulinemia by controlling the expression of enzymes involved in fatty acid and triacylglycerol synthesis, lowering SREBP-1 expression, and normalizing the mRNA level of TNF-α in the visceral adipose tissue [197]. Another study showed that cyaniding 3-glucoside is involved in the improvement of adipocytokine (leptin and adiponectin) secretion and upregulation of adipocyte-specific gene expression in rat and human adipocytes [81]. Bilberries are one of the richest sources of anthocyanins and bilberry extract (BBE) improved hyperglycemia and insulin sensitivity in diabetic mice by targeting AMPK, GLUT4, and metabolic enzymes [82]. BBE upregulated total AMPKα and the phosphorylation of AMPKα at Thr 172 and subsequently increased GLUT4 [198]. The adipocytokine retinol-binding protein 4 (RBP4) was also found to be involved in the anti-diabetic effect of anthocyanins [83]. Anthocyanins also improved insulin signaling by exciting insulin receptor (IR) phosphorylation by increasing tyrosine kinase activity in the β-subunit of the IR [83]. Additionally, anthocyanins enhanced β-cell viability and improved cellular activity by protecting islet cells against apoptosis through upregulation of Bcl-2 proteins, downregulation of Bax, and cleavage of caspase 3 proteins in diabetic rats [83]. Cyanidin-3-glucoside also protected hepatocytes against high glucose (HG)-stimulated damage by reducing the mitochondria-mediated apoptotic pathway and improving antioxidant status by triggering AKT and inactivation of JNK [84,199]. Cyanidin-3-glucoside alleviated macrophage infiltration in the adipose tissue as well as reduced the levels of mRNA of MCP-1, IL-6, and TNF-α, and phosphorylation of FOXO1 through the AKT-dependent pathway [200]. Cyanidin-3-glucoside also showed protective effects against hydrogen peroxide-induced cell death, mitochondrial ROS production, and cell necrosis against oxidative stress-induced pancreatic β cell damage [201]. Taken together, anthocyanins and their glycosides alone or in combination may repress white and brown adipose tissue weights, normalize visceral adipose tissue, and enhance glucose homeostasis by influencing β-cell mass and function, insulin sensitivity, glucose uptake, and insulin signaling.

Theaflavins are formed from the condensation of flavan-3-ols and are found in black teas, which are the world’s most popular beverages containing a set of natural polyphenol pigments [152]. Theaflavins are classified as theaflavin (TF), theaflavin-3-gallate (TF3G), theaflavin-3′-gallate (TF3′G), and theaflavin-3,3′-digallate (TF3DG). Several studies have confirmed that black tea consumption can reduce the risk of total and LDL cholesterol [202]. Black tea reduces intestinal cholesterol absorption through its inhibitory effect on pancreatic lipase activity, and TF3G was reported to have an inhibitory effect on cholesterol incorporation and hypertriacylglycerolemia [203]. Theaflavin administration at doses of 100 and 200 mg/kg of body weight suppressed hypertriacylglycerolemia in rats in a dose-dependent manner. Furthermore, the galloyl moieties of TF3G, TF3′G, and TF3DG, were found to be involved in inhibitory action on pancreatic lipase proportionately to the dose used [203].

Morin, a natural flavonoid found in almonds and other plants in the *Moraceae* family, also shows numerous health benefits by preventing obesity and diabetes [204,205,206]. Oral administration of morin for 30 days in animal models significantly enhanced hyperglycemia, glucose intolerance, and insulin resistance. Morin treatment improved the antioxidant ability and decreased lipid peroxides in diabetic rats, thus normalizing the serum lipid and lipoprotein profile. In diabetic animals, morin treatment reduced the elevation of inflammatory cytokines, including IL-1β, IL-6, and TNF-α [207]. Morin impaired the hepatic SphK1/S1P signaling pathway and ameliorated high fructose-induced reduction of hepatic NF-κB activation, subsequently decreasing the levels of IL-1β, IL-6, and TNF-α in the rat liver and BRL3A cells. Administration of morin was reported to improve hepatic insulin and leptin sensitivity, followed by subsequent decreases in blood lipid and liver lipid accumulation [208]. As an inhibitor of protein-tyrosine phosphatase 1B (PTP1B, also known as tyrosine-protein phosphatase non-receptor type 1), dietary morin sensitized and activated insulin receptor-mediated metabolic pathways [209]. Moreover, morin significantly reduced the levels of blood glucose, G6Pase, and fructose 1,6-diphosphatase (FDPase, also known as fructose 1,6-bisphosphatase) and increased the levels of insulin, hexokinase and G6PD (or G6PDH) [210].

Wogonin, a conventional herbal medicine which has long been used in East Asian countries, was also reported to have anti-obesity and anti-diabetic effects [211]. Wogonin was extracted from the root of *Scutellariabaicalensis gerogi* (*Scutellariae radix*) and was found to modulate lipid metabolism, blood glucose level, and insulin sensitivity by selectively activating PPARα and AMPK without any detrimental side effects such as weight gain or fatty liver. Pretreatment with wogonin remarkably attenuated HG-induced vascular permeability, monocyte adhesion, cell adhesion molecule expression, ROS formation, and NF-κB activation [212].

## 5. Conclusions

The prospect of using natural products to treat obesity and diabetes has not been widely examined. Flavonoids are a potential alternative treatment strategy for the development of effective and safe anti-obesity and anti-diabetes drugs. Emerging studies have described the promising role of flavonoids in treating obesity and diabetes as well as their associated metabolic diseases. The anti-obesity and anti-diabetic potential associated with flavonoids are very large given their regulatory effects on blood sugar transporters by increasing insulin secretion, reducing apoptosis, promoting pancreatic β-cell proliferation, and reducing insulin resistance, inflammation, and oxidative stress in the muscle. Determining the molecular mechanisms involved in glucose and lipid metabolism in obesity and diabetes would provide insight into the field of drug development, and future discoveries are expected to yield therapeutic benefits. With the rapidly increasing incidence of obesity and diabetes worldwide, there is a great need for safe and effective functional biomaterials with anti-obesity and anti-diabetic activities. Therefore, additional studies are needed to promote the development of nutritional flavonoids for treating obesity, diabetes, and their complications.

## Figures and Tables

**Table 1:** Representative flavonoids showing anti-obesity and anti-diabetic effects.

Name of Flavonoids	Structures	Plant Sources	Anti-Obesity and Anti-Diabetic Effect in *in Vitro*/*in Vivo* Model	Molecular Mechanism in Obesity and Diabetes	References
Quercetin	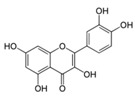	Apples, berries, red onions, cherries, broccoli, coriander, *etc*.	↑ Apoptosis in 3T3-L1 preadipocytes	↓ PARP, ↑ AMPK, ↑ Caspase 3 and 9	[45]
			↑ Glucose uptake in rat adipocyte, C2C12 muscle cells	↑ GLUT4	[46]
			↑ Glucose uptake, ↓ Fat accumulation in 3T3-L1 preadipocytes	↓ PPARγ1	[47]
			↓ Hyperglycemia, ↑ Insulin in STZ-induced diabetic rats, *db*/*db* mice	↓ NF-κB, ↓ Caspase 3, ↓ MDA levels, ↑ SOD and CAT	[48]
Rutin	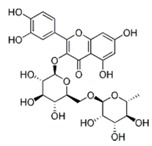	Buckwheat, oranges, grapes, lemons, limes, peaches and berries	↓ Blood lipids, ↓ Fatty liver in DIO mice and rat	↓ PPAR and ↓ C/EBP, ↓ TNF-α, ↓ IL-6	[49]
			↑ Glucose uptake in the rat soleus muscle	↑ PI3K, ↑ MAPK	[50]
Isorhamnetin	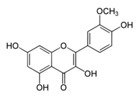	Ginkgo biloba L., *Hippophae rhamnoides* L. and *Oenanthe javanica* (Blume)	↓ Hyperglycemia and oxidative stress in STZ-induced diabetic rat, Inhibition adipogenesis in 3 T3-L1 cells	↓ PPARγ, ↓ C/EBPα	[51]
			↑ Insulin secretion in HFD-induced C57BL/6 mice	↑ GLUT2, ↑ PPARγ	[52]
Kaempferol	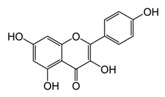	Grapefruit, tea, cruciferous vegetables	↓ Hyperglycemia, ↑ Glucose uptake in rat soleus muscle	↓ Caspase 3	[53]
			↑ β-cell survival in INS-1E cells	↑ GLUT4, ↑ AMPK	[54]
			↑ Antioxidant defense and body weight gain in diabetic rats and HFD-obese mice	↓ PPARγ, ↓ SREBP-1c, ↓ TNF-α, ↓ IL-6	[55]
Resveratrol	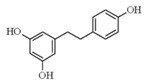	Red grapes, red wine, peanuts, and ground nuts	↑ Glucose uptake	↑ GLUT4	[56]
			↓ Lipid accumulation 3T3-L1	↓ PPARγ	[57]
			↑ Lipolytic activity in adipocytes	↑ cAMP	[58]
Naringenin	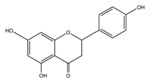	Spreng, Grapefruits, oranges and tomatoes	↓ Blood lipids, ↓ Fatty liver in Hypercholesterolemic rats	↓ HMG-CoA, ↓ ACAT	[59]
			↓ Glucose uptake in 3T3-L1 adipocytes	↓ PI3K, ↓ AKT	[60]
			↓ Hyperglycemia in STZ-induced rat	↑ Antioxidant enzyme (SOD)	[61]
Naringin	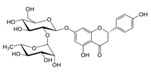	Citrus fruits and Grapefruit	↓ Hyperglycemia, ↑ Plasma insulin, ↑ Leptin in STZ-induced diabetic mice and *db*/*db* mice	↑ GLUT4, ↑ PPARγ	[59]
			↓ Blood lipids, ↓ Fatty liver in *db*/*db* Mice	↓ HMG-CoA, ↓ ACAT	[62]
Hesperidin	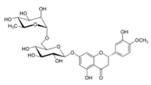	Lemons and oranges	↓ Blood glucose level, ↓ Blood lipids in STZ-induced type 1 diabetic rats	↑ Glucokinase	[63]
			↓ Oxidative stress, apoptosis	↑ GLUT4, ↓ HMG-CoA, ↓ ACAT	[64]
Eriodictyol	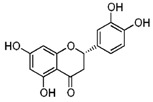	Lemon fruits	↓ Adipocyte-specific fatty acid binding protein in differentiated 3 T3-L1 adipocytes	↑ PPARγ	[65]
			↑ Glucose uptake, ↑ Insulin resistance in HepG2 cells	↑ AKT	[65]
			↓ Diabetes-related lipid peroxidation	↓ TNFα, ↓ ICAM-1, ↓ VEGF	[66]
Genistein	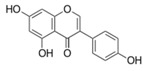	Soy foods	↓ Plasma triglycerides in Sprague-Dawley rats	↑ GLUT4	[67,68,69]
			↑ Insulin-positive β cell in HG-induced diabetic mice	↑ cAMP signaling, ↑ PKA activation	[70]
			↓ Blood glucose, ↓ Blood HbA1c in STZ-induced diabetic mice, ↓ Adipocyte differentiation	↓ TNF-α, ↓ TGFβ1, ↓ NF-κB, ↑ AMPK, ↑ ACC	[71]
Daidzein	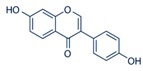	Soy foods and nuts	↓ Blood glucose, ↓ Urinary glucose	↓TNF-α, ↓ TGFβ1, ↓ NF-κB	[70]
			↓ Plasma triglycerides in Sprague-Dawley rats	↑ GLUT4, ↓ G6Pase, ↓ PEPCK	[67]
Apigenin	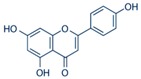	Passion flower and chamomile	↓ Hyperglycemia, ↓ Oxidative stress in STZ-induced diabetic rats and mice	↓ NF-κB, ↓ TNF-α, ↓ IL-1β	[72]
			↑ Glucose uptake, ↑ Insulin secretion in alloxan-induced diabetic mice and INS-1E cells, ↓ Lipid accumulation, ↓ Hyperglycemia in HepG2 hepatocytes	↓ G6Pase, ↑ GLUT4, ↑ AMPK, ↓ MCP-1, ↑ AMPK, ↑ ACC	[73]
Luteolin	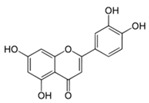	Celery, parsley, broccoli, onion leaves, carrots, peppers, cabbages, apple skins, and chrysanthemum flowers	↑ Insulin secretion in 3T3-L1 hepatocyte	↑ GLUT4, ↑ Leptin	[74]
			↑ Insulin secretion in uric acid damaged pancreatic β-cells	↓ MAFA, ↓ NF-κB, ↓ CREB-B	[75]
Tangeretin	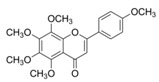	Citrus fruit rinds, mandarin orange	↑ Insulin secretion, ↑ Glycogen, ↓ Total cholesterol in HFD-induced obese mice	↓ TNF-α, ↓ IL-6, ↓ IL-1β	[76]
			↓ Plasma glucose level, ↓ Plasma HbA1c in diabetic rats	↑ AMPK	[77]
Epicatechin Gallate	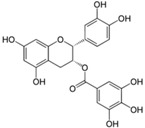	Tea, grapes and seeds of certain leguminous plants	↓ Hepatic lipid accumulation in HepG2 cells	↓ Fatty acid synthase, ↓ ACC1	[78]
(−)-Catechin	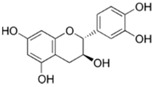	Tea, grapes and seeds of certain leguminous plants	↓ Insulin-dependent glucose uptake, ↑ Adiponectin protein	↓ KLF7, ↓ PPARγ, ↓ C/EBPα	[79]
(−)-Epigallo catechin gallate	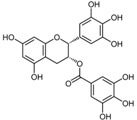	Tea, grapes and seeds of certain leguminous plants	↑ Insulin secretion, protect insulin-producing β-cells	↑ FOXO1, ↑ PDX-1, ↑ IRS2, ↑ AKT, ↑ NeuroD, ↑ MAFA	[80]
Cyanidin	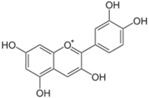	Plants with purple corn color (PCC)	↓ White and brown adipose tissue weights, ↓ Hyperglycemia	↓ TNF-α, ↓ SREBP-1	[81]
Anthocyanins	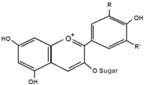	Black soybean seed coats bilberries	↓ Hyperglycemia, ↑ Insulin sensitivity, ↑ GLUT4 (WAT and muscle) in T2DM mice	↑ AMPK, ↓ PEPCK, ↓ G6Pase, ↓ ACC1, ↓ PPARα, ↑ Acyl-CoA oxidase, ↑ CPT-1A, ↓ RBP4	[82]
Cyanidin-3-glucoside	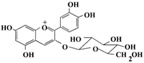	Plant bayberry fruit	Protect hepatocytes ↓ HG-stimulated damage	↑ AKT, ↓ JNK, ↓ TNF-α, ↓ IL-6, ↓ MCP-1	[83]
			↑ Insulin secretion in oxidative stress-induced pancreatic β damage	↑ GLUT4, ↑ LPL, ↑ FAS, ↑ AMPK	[84]

(↓) Decrease, (↑) Increase.
